# Measurement and Correction of Microscopic Head Motion during Magnetic Resonance Imaging of the Brain

**DOI:** 10.1371/journal.pone.0048088

**Published:** 2012-11-07

**Authors:** Julian Maclaren, Brian S. R. Armstrong, Robert T. Barrows, K. A. Danishad, Thomas Ernst, Colin L. Foster, Kazim Gumus, Michael Herbst, Ilja Y. Kadashevich, Todd P. Kusik, Qiaotian Li, Cris Lovell-Smith, Thomas Prieto, Peter Schulze, Oliver Speck, Daniel Stucht, Maxim Zaitsev

**Affiliations:** 1 Department of Radiology, University Medical Center Freiburg, Freiburg, Germany; 2 Department of Electrical Engineering, University of Wisconsin-Milwaukee, Milwaukee, Wisconsin, United States of America; 3 Biomedical Magnetic Resonance, Otto-von-Guericke University, Magdeburg, Germany; 4 Department of Medicine, University of Hawaii at Manoa, Honolulu, Hawaii, United States of America; 5 Department of Neurology, Medical College of Wisconsin, Milwaukee, Wisconsin, United States of America; University of California San Francisco, United States of America

## Abstract

Magnetic resonance imaging (MRI) is a widely used method for non-invasive study of the structure and function of the human brain. Increasing magnetic field strengths enable higher resolution imaging; however, long scan times and high motion sensitivity mean that image quality is often limited by the involuntary motion of the subject. Prospective motion correction is a technique that addresses this problem by tracking head motion and continuously updating the imaging pulse sequence, locking the imaging volume position and orientation relative to the moving brain. The accuracy and precision of current MR-compatible tracking systems and navigator methods allows the quantification and correction of large-scale motion, but not the correction of very small involuntary movements in six degrees of freedom. In this work, we present an MR-compatible tracking system comprising a single camera and a single 15 mm marker that provides tracking precision in the order of 10 m and 0.01 degrees. We show preliminary results, which indicate that when used for prospective motion correction, the system enables improvement in image quality at both 3 T and 7 T, even in experienced and cooperative subjects trained to remain motionless during imaging. We also report direct observation and quantification of the mechanical ballistocardiogram (BCG) during simultaneous MR imaging. This is particularly apparent in the head-feet direction, with a peak-to-peak displacement of 140 m.

## Introduction

Magnetic resonance imaging has become an indispensable tool for imaging of the human brain, both in patients for clinical diagnosis and in healthy volunteers for research purposes. There is a general trend to higher magnetic field strengths, which improves the image signal-to-noise ratio and therefore allows higher nominal resolution [1]. However, with higher resolution imaging, the requirement for patients or subjects to remain motionless increases. This problem worsens with the longer scan durations required to achieve the highest resolutions. The result is that motion often limits the effective resolution. Large motion is typically observed in the youngest and oldest patients, rendering the images non-diagnostic. However, even in cooperative volunteers, image quality can be degraded by small involuntary motion, such as that due to breathing, swallowing or the cardiac cycle.

Traditional means of preventing motion artifacts have major disadvantages. Sedation is expensive, not entirely effective, invasive, and unacceptable for research. Restraints are also not entirely effective and may increase the patient's feeling of claustrophobia. Self-navigating methods, such as PROPELLER (Periodically Rotated Overlapping ParallEL Lines with Enhanced Reconstruction) [2], are popular, but are specific to particular imaging sequences and often increase scan times. A promising alternative is prospective motion correction [3]–[7]. Head motion is tracked in six degrees of freedom (DOF) and the scanner pulse sequence is adjusted in real time, so that the imaging volume follows the head motion. Ideally, tracking should be performed independently from the MR scanning process, since no extra scan time is then needed to obtain position information, and the magnetization is unaltered [3]. Prospective motion correction has several advantages over retrospective techniques: it can be applied to most common pulse sequences; it ensures that the object remains in the field of view; adequate and homogeneous sampling of *k*-space is maintained; and it prevents spin history effects [8]. The method is able to correct for large-scale movements of several millimeters/degrees or more [3].

Prospective motion correction has obvious potential for routine clinical imaging, where patients may have difficulty remaining still, and this is the likely application of promising new methods, such as PROMO (PROspective MOtion correction) [6]. We hypothesize that the same general technique could also be applied in high-resolution imaging of cooperative subjects to improve the effective resolution. Existing prospective motion correction implementations, however, are limited by the ability to measure head motion with sufficient accuracy, precision, and speed. Previous work has indicated that tracking precision must be several times better than the desired image resolution, or artifacts will result from the ‘pseudo motion’ introduced by the tracking noise [9].

The goal of this work was to develop an optical tracking system to allow the correction of very small motion at a range of commonly used field strengths. For this purpose, we have further developed a system originally conceived for motion capture in biomechanics. The system, which we call moiré phase tracking (MPT), uses a passive marker that generates moiré patterns to quantify out-of-plane rotations from a single image [10], [11]. The MPT marker consists of three layers, constructed by printing planar gratings of different spatial frequencies on either side of a transparent substrate. The layers generate moiré patterns, and the phase of these patterns depends on the marker orientation. The phase, and hence the through-plane orientation of the marker, is computed using a curve fitting algorithm. The other four parameters required to define the pose in six degrees of freedom (DOF) are determined using conventional photogrammetric techniques. The marker is backed with a retroreflective material to reduce the lighting requirements of the camera and lighting unit (CLU).

An early version of the MPT system was previously applied to prospective motion correction in MRI, but this system was not fully MR compatible. The camera had to be placed outside the scanner bore at 2 to 5 m from the marker (depending on the MR scanner used), which prevented high-precision tracking [12]. In addition to the camera unit, an external mirror was required to allow a view of the marker, which is impractical for daily use. In this work, we report technical developments that enable the system to be used as a high-precision head tracking tool in MRI. This includes a reduction in the marker size, computational improvements that enable higher frame rates in real time, and importantly, the development of an in-bore MR-compatible camera and lighting unit with properties suitable for photogrammetry. We tested the function and MR compatibility of the system at three common field strengths (1.5 T, 3 T and 7 T) and applied in vivo prospective motion correction using the CLU.

## Results

**Figure 1:**
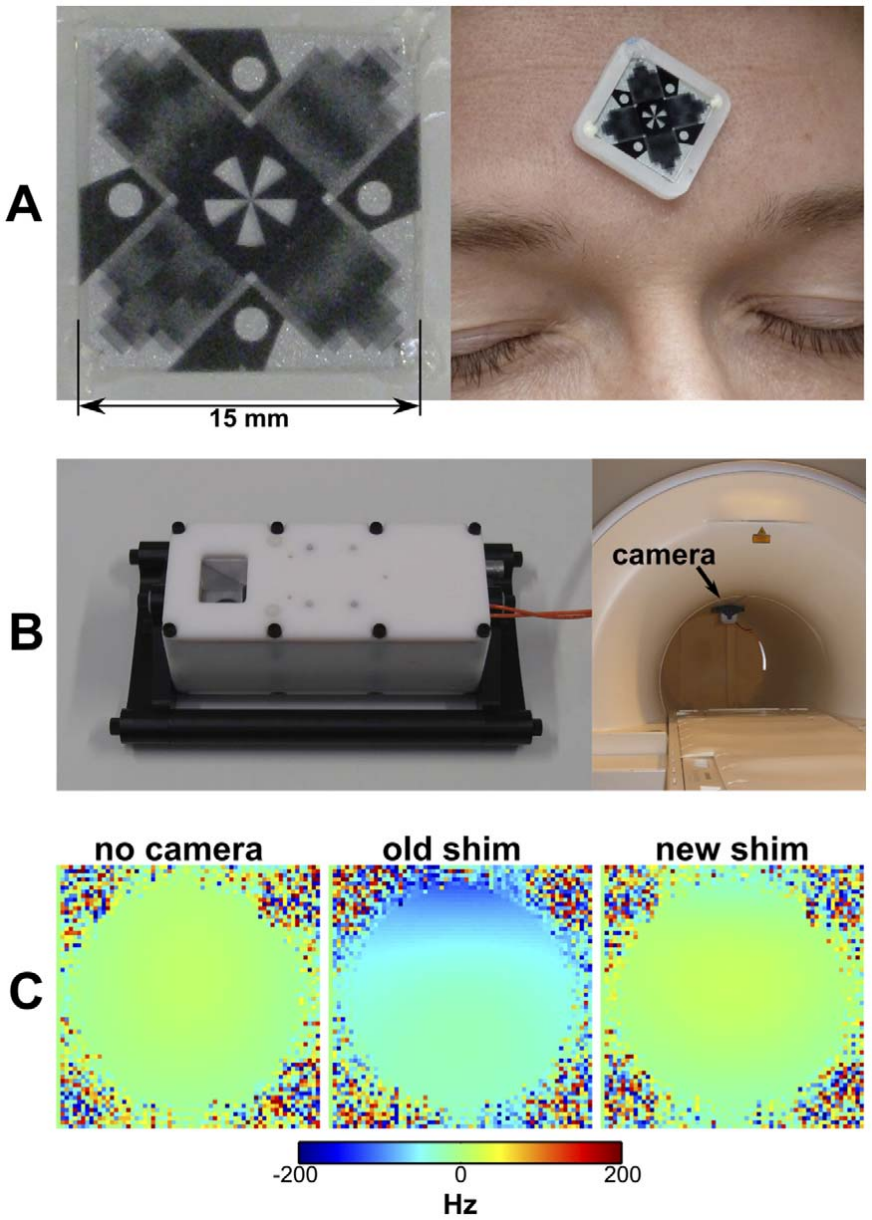
The 15 mm MPT marker (A) and the camera and lighting unit (B) developed in this work. Out-of-plane rotations are quantified using the moiré patterns generated by the marker (see Video S1). In (A) the marker is shown mounted on the head of a subject. The image contrast is modified to enhance visibility of the moiré patterns. Part (C) shows field maps acquired in a water phantom at 3 T without the camera (left), with the camera, but without reshimming (middle), and with the camera, but after a new shim (right).

The MPT system comprises a planar tracking marker, a single camera, and software to process the camera images to compute pose (position and orientation) in six degrees of freedom. The design and construction of each these components are described in *Materials and Methods*. Figure 1A shows the MPT marker. Translations and in-plane rotations are calculated using conventional computer vision techniques; out-of-plane rotations are computed using the moiré patterns generated by parallel lines printed on opposite sides of a transparent sheet (see Video S1). The marker is then attached to the forehead of the subject (as shown) using medical-grade tape or attached to a custom-made mouthpiece for more rigid coupling to the skull. The camera and lighting unit (Fig. 1B) contains a light-emitting diode, which illuminates the MPT marker. The CLU is attached inside the scanner bore using adhesive strips, allowing a direct line of sight to the marker (shown here in a 3 T Siemens Trio). The depth of field for tracking purposes is around 60 cm.

Results of RF-compatibility tests (sweeping the entire receive bandwidth for each MR system under test) showed that CLU increases total background RF noise by 0.1% at 3 T and 7 T and 1.1% at 1.5 T, which, although statistically significant in all cases (p<0.05), is negligible from a practical point of view. No structured noise was apparent at 3 T and 7 T, indicating no narrowband interference at any frequency in the MR receive range. At 1.5 T, some narrowband interference was detected; however, this effect is not strong enough to be visibly apparent in any in vivo or phantom images acquired to date. The CLU slightly affects magnetic field (B0) homogeneity, although this effect can be largely corrected by reshimming (Fig. 1C). No camera-related artifacts were visible in any of the MR images obtained while using the system.

In the following, precision and accuracy of the tracking system are specified using a coordinate system defined by the gradient fields of the MR scanner. We refer to the patient left-right axis for a patient lying supine as *x*, the vertical axis as *y*, and the axis that runs in the direction of the scanner bore as *z*. With the camera attached to the top of the magnet bore, as shown in Fig. 1B, the y axis then corresponds to through-plane motion of the marker, and is the direction of the lowest precision and accuracy. Precision, as defined by the standard deviation of tracking noise for a stationary marker during MR imaging, is 1 m, 12 m and 1 m in the scanner x, y and z directions, respectively, and better than 0.01° in all three rotations. Drifts are negligible (<1 m/minute, <0.005 degrees/minute) if the CLU is powered on for more than 80 minutes, so as to maintain a constant temperature. Tracking accuracy, measured using a rotary table outside of the scanner, but with comparable camera-marker geometry and a motion range of 360° in plane and through-plane tilts of up to 49°, is 0.7 m, 106 m and 0.7 m in the scanner x, y and z directions, respectively, and better than 0.07° in all three rotations (one standard deviation).

**Figure 2:**
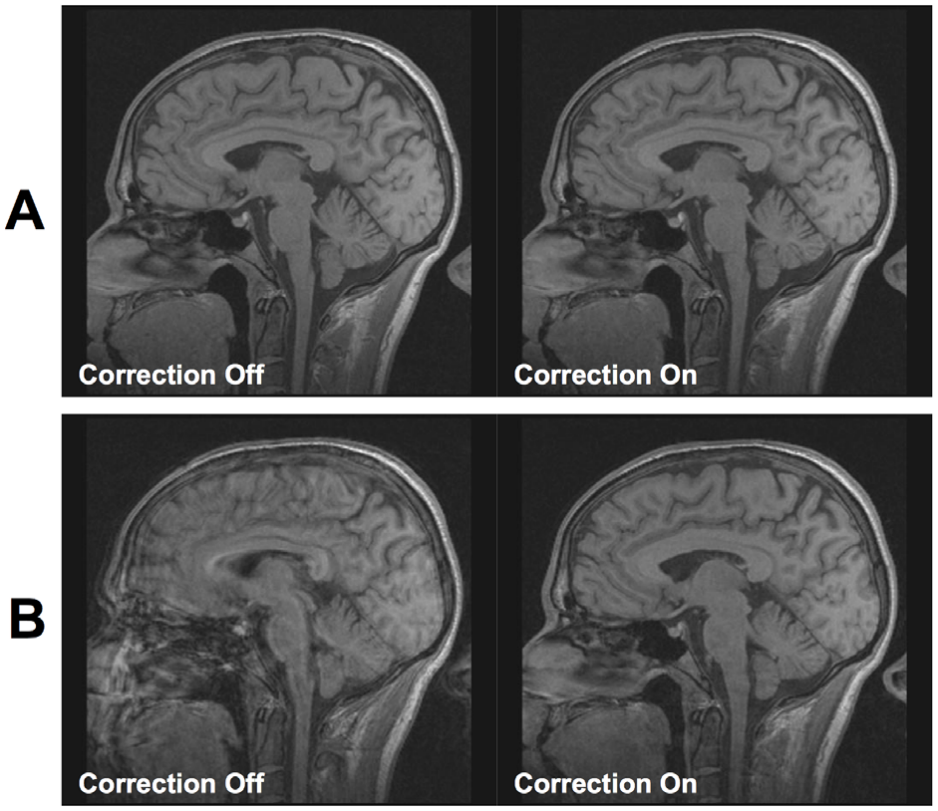
Gradient echo images obtained at 1.5 T, with an in-plane resolution of 1 mm×1 mm and a through-plane resolution of 2 mm. (A) Motion correction applied with no deliberate motion using MR imaging. (B) Motion correction when the subject performed deliberate rotations every 30 seconds during the scan when instructed by the scanner operator (motion range was approximately 8° and 25 mm). At this resolution, motion correction produces a noticeable improvement only in the case of larger movements. See Fig. S1 for a larger version of these images, along with motion plots.

Imaging results obtained at 1.5 T using a gradient echo sequence are shown in Fig. 2. In the case of no deliberate motion (Fig. 2A), there is no visible difference in image quality with or without correction. Results with deliberate motion, however, demonstrate that the method can improve image quality at this field strength in cases of larger motion (Fig. 2B). Motion plots quantifying the motion that occurred in each case were similar (about 8° and 25 mm for Fig. 2B) and are given in Fig. S1.

**Figure 3:**
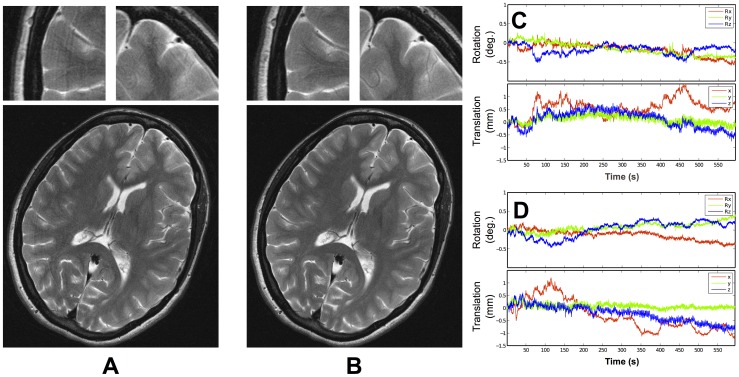
TSE images obtained at 3 T, with an in-plane resolution of 0.3 mm×0.3 mm and a slice thickness of 3 mm. (A) without motion correction; (B) with motion correction. The subject tried to remain as still as possible in both cases. Motion plots from (A) and (B) are shown in (C) and (D), respectively.

Figure 3 shows results at 3 T using a turbo spin echo (TSE) sequence (0.3 mm in-plane resolution) with a subject with no intentional motion. Both a reduction in ghosting artifacts and an improvement in visibility of fine structures are apparent. In this example, the MPT marker was attached to the skin, as shown in Fig. 1.

**Figure 4:**
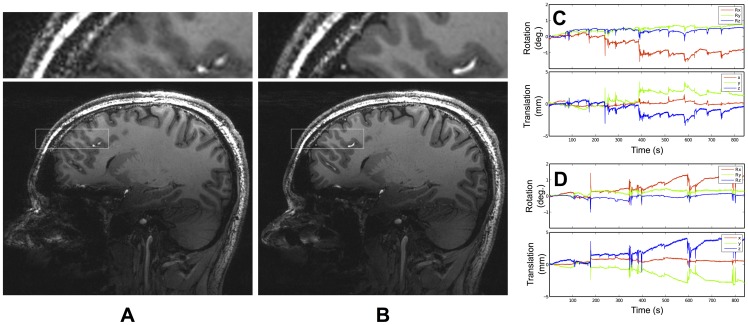
MP-RAGE images obtained at 7 T, with an isotropic resolution of 0.6 mm. (A) without motion correction; (B) with motion correction. The subject tried to remain as still as possible in both cases. Motion plots from (A) and (B) are shown in (C) and (D), respectively. All slices from (A) and (B) can be seen in the supporting information (Video S2 and S3, respectively).

Figure 4 shows results at 7 T (MP-RAGE sequence). Image quality is visibly improved when prospective correction is used. The tracking data indicate that the subject, although well trained, inadvertently moved his head by several millimeters/degrees during both 14-minute scans. In our experience, this gradual drift in pose is typical with such long scan durations. Additionally, some large transient spikes are visible, which are likely due to the subject swallowing. This problem is aggravated by the use of the mouthpiece, as it encourages saliva build-up.

**Figure 5:**
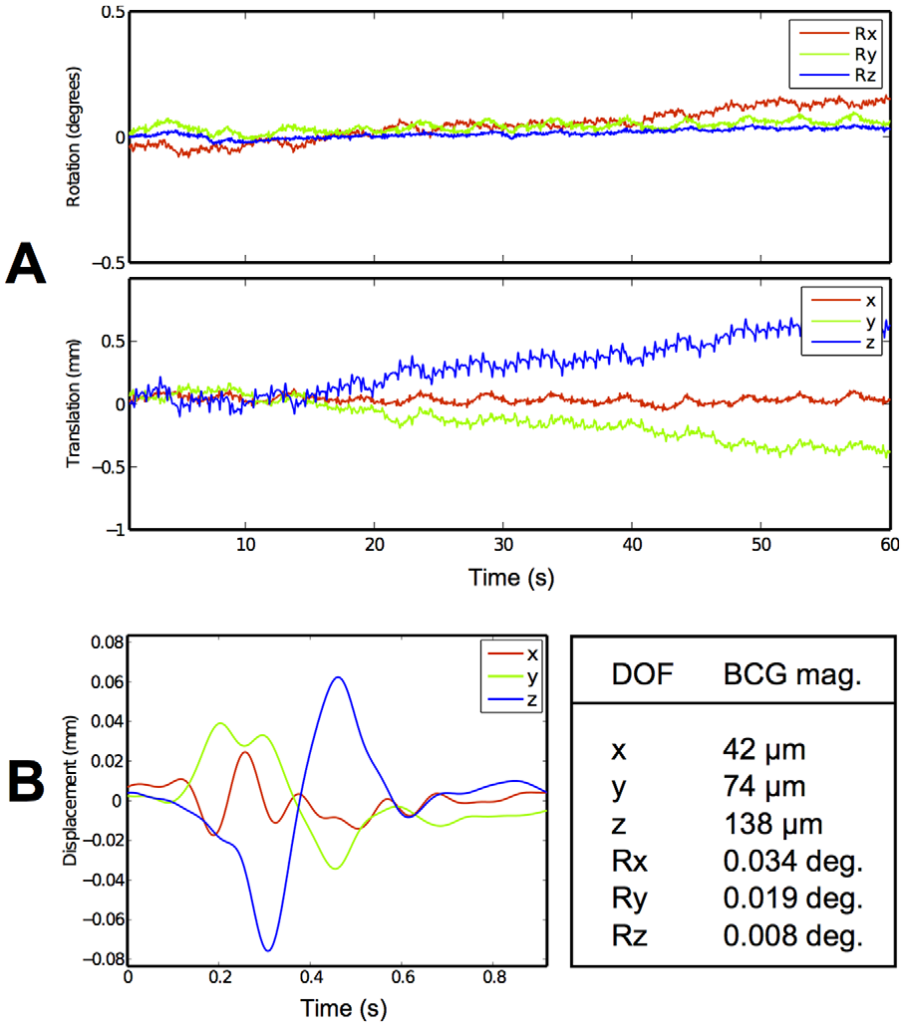
Head tracking data from the experiment at 7 T showing subject motion during simultaneous MR imaging. (a) cardiac and respiratory components are visible in the unprocessed data (see further examples in Fig. S2); (b) a ballistocardiogram formed by zero-phase filtering, peak detection, and ensemble averaging of 639 beats in the original (full length, unfiltered) signal. The head-feet DOF shows the effect most strongly, with a peak displacement of 138 m in this example. Similar ballistocardiograms were obtained from the experiments performed at 1.5 T and 3 T (see Fig. S3).

Figure 5 shows the tracking data from the first minute of the second scan in the 7 T experiment (Fig. 4B). Tracking data show cardiac-related head motion, most prominently in the head-feet direction. This effect is apparent in all data collected using the system, regardless of the marker fixation method used (see further examples in Figs. S2 and S3). Breathing motion is also often apparent.

## Discussion

Several other groups have successfully applied prospective motion correction using an external tracking system (e.g., [4], [5], [7]). The work reported here extends these previous studies by greatly increasing the precision and accuracy of tracking data and applying prospective correction during MRI of normal subjects to correct for tiny involuntary movements. The MR-compatible tracking system developed in this work builds on several previous designs, including an off-the-shelf non-MR-compatible stereo camera system [3] and an ‘out-of-bore’ single-camera MPT system [12], [13]. These were useful for proof-of-concept studies, but required an unobstructed view into the magnet bore, making them impractical. Our initial in-bore designs (e.g., that reported in [14]) were not sufficiently MR compatible. The work reported here avoids all problems encountered in these earlier designs.

When the CLU is installed in a new scanner, an initial scanner-camera cross-calibration procedure must be performed. This typically takes 1–2 hours, including the time required to mount the camera on the inside of the scanner bore. We are currently testing several faster methods for calibration, but these were not used in the experiments described in this work. Regardless of the calibration method used, the procedure does not need to be repeated unless the camera is moved, since no special recalibration is required when changing subjects. It is therefore of great benefit if the CLU can be left inside the scanner permanently. To facilitate this, the size of the unit should be as small as possible. The current CLU dimensions are 7×7×17 cm; however, there is significant potential for size reduction. In particular, the charge pump circuitry takes up a large portion of the CLU volume and could be miniaturized.

The MR compatibility of the CLU was verified on 1.5 T, 3 T and 7 T systems. In all cases, the CLU was unaffected by the magnetic fields of the scanner. RF testing was performed at all field strengths, since the receive frequency of the scanner is proportional to the field strength and thus RF interference could occur on one system but not on others. In no case were problems observed, which indicates that the 40 m copper shielding is effective. Field distortions were measured at 3 T. After reshimming, the field homogeneity is acceptable for imaging. Further improvements could be achieved by reducing the size of the CLU, since this would reduce the amount of field-distorting material present.

The precision of tracking data during simultaneous MR imaging represents a one to two order of magnitude improvement over previously reported commercial or custom-built systems [3], [7], [12]. When testing an early version of the in-bore camera, we experienced problems with vibrations caused by eddy currents induced in the aluminum shielding used at that time. The much thinner copper shielding used here avoids this problem. The lack of RF interference indicates that the current copper shielding represents a good compromise between RF attenuation and avoiding eddy current-related vibrations. Earlier theoretical work [9] indicates that the level of precision obtained by the system is more than good enough for motion correction at imaging resolutions of better than 100 m. The latency (lag) time, estimated to be 39 ms at a frame rate of 80 Hz, is related to the buffering and processing of camera images and is an area of possible improvement. Given that the peak-to-peak displacement of 140 m shown for cardiac-related head motion (Fig. 5B) occurs in only 150 ms, applying coordinate updates with pose data that is 39 ms old means that a relevant portion of this motion remains uncorrected. If the latency cannot be reduced, a further option could be predictive filtering or correction of the residual motion in post-processing [15]. Nonetheless, the speed of the system presented here is an improvement over our previous work, where a ‘data rejection and reacquisition’ strategy was required, due to inadequate correction under rapid motion [13]. With the tracking system described here, this approach is no longer necessary, and was not used in this work.

In the experiments at 1.5 T, the correction of the large, deliberate motion is clearly the cause of the improvement in the images shown in Fig. 2B. It is unsurprising that no imaging quality improvement is seen in Fig. 2A, since the resolution is relatively low (1 mm) and therefore slight motion is not expected to cause noticeable degradation of image quality. At 3 T and 7 T, however, it is unclear which component of motion (cardiac, breathing or involuntary muscle relaxation) is most responsible for the improvement in image quality shown with correction. Determining this is an important area for future studies, which should also independently record cardiac and respiratory motion using other measures. Such a study should ideally also seek to provide statistical validation of the results on a large number of subjects. The artifacts prevented by prospective motion correction could also be simulated using retrospective motion correction or reproduced using the method mentioned in [16]. However, these methods would not take secondary effects such as motion-related B0 field changes into account, so validation with a large group of subjects will be essential.

Similarly, further work should be performed to determine the possible implications for research or clinical imaging resulting from the gains in image quality observed. The preliminary results suggest an improvement in effective image resolution (through reduction of motion-induced blurring) given identical nominal resolutions, particularly at very high resolution; however, quantifying the degree of improvement would be a useful first step. For standard clinical routine imaging, a reduction or even complete prevention of the need for repeat scans or examinations is expected, leading to a significant improvement in diagnostic quality, clinical workflow, patient compliance and cost savings.

Prospective motion correction compensates for the changing orientation of the encoding fields relative to the moving subject, but a number of secondary motion-induced effects remain uncorrected. Examples include B0 field distortions [17], [18] and corruption of Nyquist ghost correction in EPI [19]. These issues, and others, may result in residual artifacts in the case of large motion. However, the motion under consideration here is so small that these effects are likely to be less significant than errors caused by poor marker fixation. Although good results were obtained here with our well-trained volunteers, skin motion unrelated to motion of the head could potentially corrupt the tracking data, so alternative methods of attaching the marker need to be examined. Finally, it is important to note that we correct for only rigid-body motion in this study. Non-rigid brain pulsations, reported to reach about 100 m in displacement magnitude [20], are not corrected for.

To our knowledge, this is the first time that a ballistocardiogram has been produced in a full six DOF during simultaneous MR imaging. Motion of up to 140 m may be of increasing importance as field strengths and nominal resolutions increase. In addition, the method of ensemble averaging we use for determining the ballistocardiogram may slightly underestimate the peak-to-peak magnitude if the period is not constant. The observed BCG is consistent with ballistocardiography literature, such as Ref. [21]. Displacements in the head-feet direction are reportedly due to the ejection of blood from the heart. The result indicates potential for respiratory and cardiac gating based on motion tracking data. This could be of particular interest at high field (e.g., 7 T), where the ECG signal is highly distorted and gating is problematic. Signal quality after application of the bandpass filter is very high, and MPT data might provide a more robust means of gating than ECG at high fields [22]. It is also important to note that the peaks observed are related to, but not the same as, the QRS complex observed in ECG. Simultaneous head tracking and ECG recording would reveal more about this, including their relative timing.

In conclusion, we have developed an MR-compatible tracking system with precision on the order of 10 m during simultaneous MRI. This is far more precise than existing systems, thus opening up new applications. We have used the system to quantify head motion induced by the respiratory and cardiac cycles, as well as other movements, during MR imaging. Preliminary results with three subjects indicate substantial improvements in image quality, both for intentional and unintentional movements, when the MPT tracking data are used for prospective motion correction. Additionally, the tracking system may have other applications whenever accurate position tracking is required in the MRI environment.

## Materials and Methods

### Ethics statement

All experiments on human volunteers were performed with the approval of the ethics committee of Albert Ludwigs University Freiburg, Germany (for 1.5 T and 3 T experiments) or the ethics committee of the Otto-von-Guericke University Magdeburg, Germany (for 7 T experiments). All subjects gave their written informed consent before the commencement of the study.

### Camera design, construction and calibration

The camera was designed by the authors for accurate head tracking during simultaneous MR imaging. To provide high-quality images for photogrammetry, specifications include a frame rate of higher than 80 frames/s; a short exposure time (normally 50–100 s) to minimize motion blur; a global shutter to prevent distortion effects when imaging a moving object; and fixed and stable optics. Camera calibration is performed using bundle adjustment [23].

To ensure MR compatibility, the CLU enclosure was constructed from acetal and lined with 40 m copper shielding to prevent RF interference while minimizing eddy-current induced vibrations. The copper shield also serves as a heat spreader to ensure adequate surface area for conduction through the acetal enclosure. Images are transferred out of the scanner room using a fiber optic IEEE 1394b connection, rather than via copper cable, to improve MR compatibility. The CLU is powered using a single DC supply via coaxial cable, grounded to the Faraday cage of the scanner room. Charge pumps are used in the CLU to provide the required voltages. On-axis marker illumination is achieved through a half-silvered mirror, built into the CLU enclosure. The optics were designed so that the system can track a marker located anywhere between approx. 5 and 65 cm from the CLU. The optical components are mounted on a ceramic block, to ensure long term stability.

Since MPT tracking is independent of the MRI scanner, the tracking data must be transformed from the camera frame of reference into the scanner frame of reference. This is a trivial operation once the correct transformation matrix is known, but initially this is not the case. For the experiments described in this work, we applied the following method to compute the transformation. An MPT marker was fixed to a phantom and the phantom was imaged while tracking data from the camera were logged to file and averaged. This procedure was repeated 10-15 times, with small (e.g., 10°) rotations applied to the phantom between each scan. The difference between consecutive poses was computed using 3D image registration (for the MRI data) and straightforward quaternion operations (for the tracking data). The transformation between the two coordinate systems was then obtained using a least-squares optimization method, such that the measured pose difference data using the two methods became as consistent as possible (after transformation to the MRI coordinate system). In the 1.5 T and 3 T experiments, this transform was then optimized further by applying the procedure described in [3]. In this procedure, the phantom is rotated by 180° with prospective motion correction enabled between scans. The resulting phantom volumes are then approximately aligned, and any residual errors, as determined by image registration, are used to refine the transform. This procedure was not performed at 7 T, due to problems with performing the 180° rotations in long-bore scanner. This one-time calibration procedure per scanner generally takes 1–2 hours. No subject-specific calibration is necessary.

### MPT marker improvements

The MPT markers were originally described for robotic and kinesiological applications [10], [24]. Lithographic methods used to make integrated circuit masks have been used to miniaturize the markers for tracking within the space constraints of MRI.

### MR compatibility testing

RF testing was performed at 1.5 T, 3 T and 7 T. In each case, the CLU was installed in its usual location in the magnet bore. Data were recorded with the MR scanner by sweeping the entire frequency bandwidth, without applying any RF or gradient pulses. This was performed twice: once with the system powered off and with the external BNC cable disconnected; and once with the tracking system powered on and tracking. Field mapping was performed at 3 T on a 16 cm water phantom placed at the magnet isocenter. The effect of imaging on CLU vibrations was measured by recording tracking data from a stationary phantom during imaging with a gradient echo sequence. This will slightly overestimate the effect of vibrations (i.e., underestimate the precision), due to vibrations of the phantom itself.

### In vivo experiments

Two identical CLU systems were used for prospective motion correction of head motion at two different sites and on three different Siemens MR systems: a 1.5 T Symphony, a 3 T Trio and a 7 T system. A different volunteer was scanned on each system. Volunteers were experienced at undergoing brain MRI and had previously demonstrated their ability to remain relatively motionless during imaging. They were instructed to stay as still as possible during the experiments. The tracking marker was fixed to the subject using one of two methods: directly to the forehead (1.5 T and 3 T experiments) or via a mouthpiece (7 T experiments). Tracking data were logged to file for later analysis. In all cases, the camera frame rate was set to 80 Hz; however, the average sampling rate of tracking data was closer to 79 Hz, since not all frames could be processed in real time (computation speed is currently the limiting factor for frame rate). All MR imaging sequences were product sequences modified by the authors to allow real-time update of the imaging volume during scan execution. All acquired MR data were used in image reconstruction (i.e., no data rejection and reacquisition strategy was employed).

At 1.5 T the subject was imaged using a 3D gradient echo sequence (TE: 4.05 ms, TR: 13 ms, flip angle: 15 deg., resolution: 1 mm×1 mm in-plane, 2 mm through-plane, acquisition time: 4 min. 40 s). Four scans were performed, initially with no deliberate motion and then with controlled motion every 30 s when instructed by the scanner operator. The motion performed consisted of alternating left-right rotations of about 8° (about the z-axis) with a corresponding shift of about 25 mm in the x-direction. The imaging volume of the two motion-corrected scans were ‘locked’ to the position of the imaging volume of the first scan, using functionality of the motion correction software developed by the authors. This allows motion-corrected images to be compared easily to non-corrected images, since the slices are then a good match.

The 3 T study was performed using a 2D turbo spin echo (TSE, [25]) sequence (TE: 87 ms, TR: 6500 ms, echo train length: 21, resolution: 0.3 mm×0.3 mm in-plane, 3 mm through-plane, acquisition time: 9 min. 45 s) and a 12-channel head coil. Two scans were performed: first without motion correction and then with motion correction. This order was deliberately chosen to avoid a positive bias for the correction, since, in our experience, subjects tend to move more towards the end of their examination.

The 7 T experiment was performed using a 3D MP-RAGE [26] sequence (TE: 2.69 ms, TR: 2500 ms, TI: 1050 ms, flip angle: 5 deg., resolution: 0.6 mm isotropic, acquisition time: 14 min. 3 s) and a 32-channel head coil. Again, two scans were performed: first without motion correction and then with motion correction. This experiment differed from the 1.5 T and 3 T experiments in that a mouthpiece was used to hold the MPT tracking marker. This was necessary to allow an unobstructed view of the marker, since the head coil used was fully enclosed.

### BCG data analysis

The ballistocardiogram (BCG) waveforms are computed using the following procedure. All head tracking data acquired during simultaneous MR imaging data are resampled at a rate of 100 Hz using linear interpolation. This greatly simplifies further processing, since although the original frame times are precisely known, they are not necessarily evenly spaced in time (occasionally a frame is dropped if the image processing rate falls behind the camera frame rate). Peaks in the tracking data are then located in the z-component of the translation data. This is done applying a zero-phase bandpass filter to the data (passband: 0.6 Hz to 10 Hz, stopbands: below 0.5 Hz and above 12 Hz, stopband attenuation: 40 dB). Peak detection is then applied to the result. Peaks are then automatically discarded if their height is more than one standard deviation from the mean height or if the distance to either of their neighboring peaks is more than one standard deviation from the mean distance. The locations of all remaining peaks are then used to identify the location of the peaks in the original (unfiltered) data in all DOF. This approach is valid, due to the zero-phase nature of the bandpass filter. These repeating segments are averaged to form the final BCG (translation part shown in Fig. 5; ensemble average of 639 beats).

## Supporting Information

Figure S1 **Gradient echo images obtained at 1.5 T, with an in-plane resolution of 1 mm×1 mm and a through-plane resolution of 2 mm.** (A) Motion correction applied with no deliberate motion using MR imaging. (B) Motion correction when the subject performed deliberate rotations every 30 seconds during the scan when instructed by the scanner operator (motion range was approximately 8 degrees and 25 mm). At this resolution, motion correction produces a noticeable improvement only in the case of larger movements. (C) Motion plots corresponding to the two scans shown in (B) indicates that the motion performed was comparable between the two experiments.
(PDF)

Figure S2 **Tracking data from (A) the 1.5 T and (B) the 3 T experiments.** In these examples, the MPT marker was attached directly to the forehead of the subject; this differs from the results obtained at 7 T, and shown in Fig. 5, where a mouthpiece was used. With both marker attachment methods (and for all three scanners and subjects), the ballistocardiogram is visible in the z direction. In the examples shown here, respiratory motion is also clearly visible.
(PDF)

Figure S3 **Ballistocardiograms computed using tracking data from (A) the 1.5 T and (B) the 3 T experiments (see** **Fig. 5** **for results obtained at 7 T).** In each case, the BCG appears most strongly as a shift along the head-feet direction.
(PDF)

Video S1 **The moving MPT marker.** Translations and in-plane rotations are calculated using conventional computer vision techniques. Out-of-plane rotations are computed using moiré patterns, as seen in this video. This enables highly accurate six-degree-of-freedom pose information to be computed from a single planar marker.
(AVI)

Video S2 **All acquired slices from the experiment corresponding to the results shown in** **Fig. 4A** **(motion correction off).** Although the images shown would be conventionally regarded as of very high quality, when compared to Video S3, the reduced clarity is obvious.
(MOV)

Video S3 **All acquired slices from the experiment corresponding to the results shown in** **Fig. 4B** **(motion correction on).**
(MOV)
