# Correction: Measurement and Correction of Microscopic Head Motion during Magnetic Resonance Imaging of the Brain

**DOI:** 10.1371/annotation/a29733ae-6317-42ee-92c0-a49542e1b7c8

**Published:** 2013-06-28

**Authors:** Julian Maclaren, Brian S. R. Armstrong, Robert T. Barrows, K. A. Danishad, Thomas Ernst, Colin L. Foster, Kazim Gumus, Michael Herbst, Ilja Y. Kadashevich, Todd P. Kusik, Qiaotian Li, Cris Lovell-Smith, Thomas Prieto, Peter Schulze, Oliver Speck, Daniel Stucht, Maxim Zaitsev

The letter "m," used as an abbreviation of "meter," appears incorrectly throughout the manuscript. The correct abbreviation should be "um," to indicate micrometer(s). 

