# Supplementary material for: Measurement and Correction of Microscopic Head Motion during Magnetic Resonance Imaging of the Brain
Source: PLoS One. 2012 Nov 7;7(11):e48088. doi: 10.1371/journal.pone.0048088 (PMC3492340; doi:10.1371/journal.pone.0048088)
Supplement: Figure S1 — Gradient echo images obtained at 1.5 T, with an in-plane resolution of 1 mm×1 mm and a through-plane resolution of 2 mm. (A) Motion correction applied with no deliberate motion using MR imaging. (B) Motion correction when the subject performed deliberate rotations every 30 seconds during the scan when instructed by the scanner operator (motion range was approximately 8 degrees and 25 mm). At this resolution, motion correction produces a noticeable improvement only in the case of larger movements. (C) Motion plots corresponding to the two scans shown in (B) indicates that the motion performed was comparable between the two experiments. (PDF) [file pone.0048088.s001.pdf]

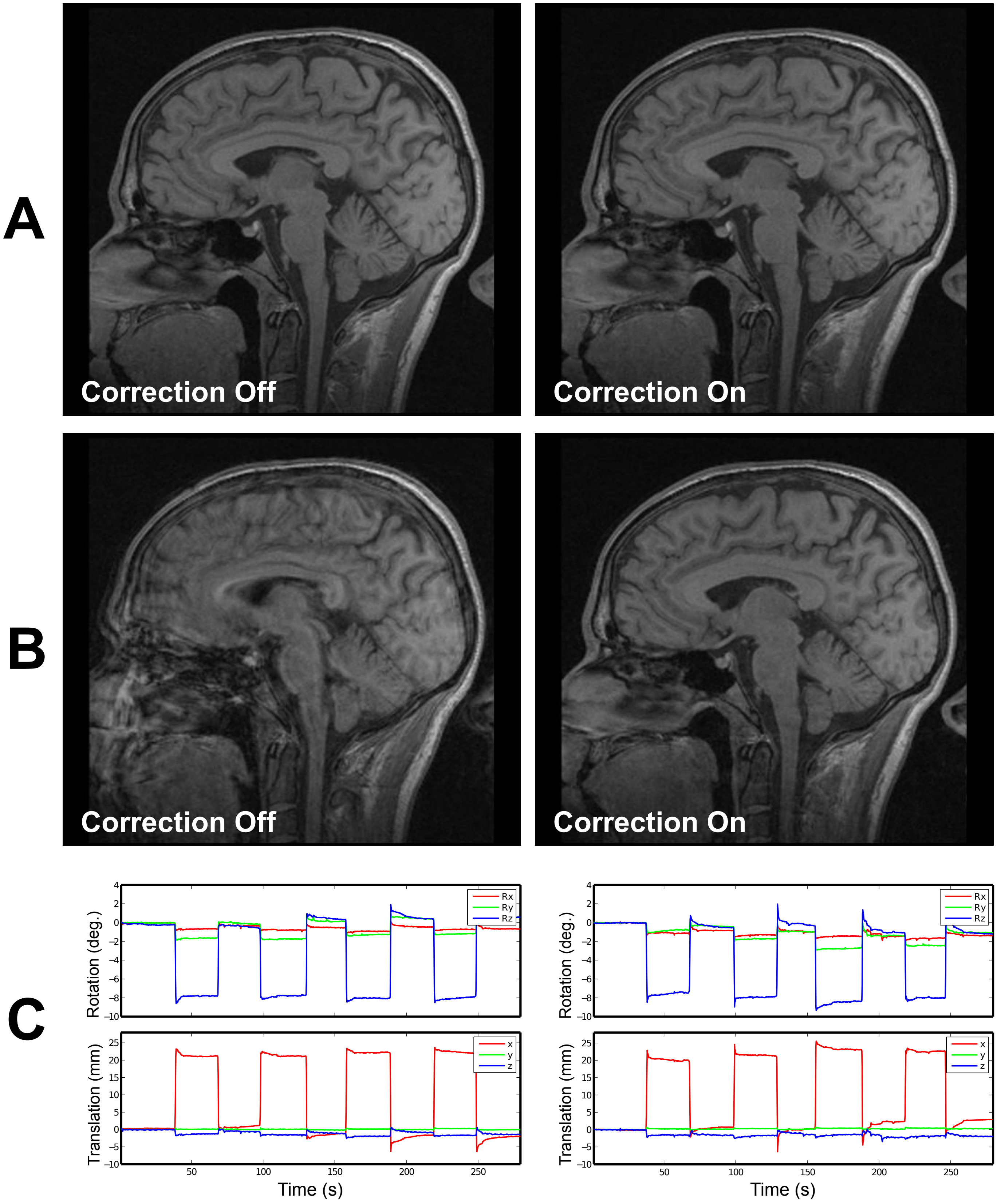

Fig. S1. Gradient echo images obtained at 1.5 T, with an in-plane resolution of 1 mm×1 mm and a through-plane resolution of 2 mm: (A) Motion correction applied with no deliberate motion using MR imaging. (B) Motion correction when the subject performed deliberate rotations every 30 seconds during the scan when instructed by the scanner operator (motion range was approximately 8 degrees and 25 mm). At this resolution, motion correction produces a noticeable improvement only in the case of larger movements. (C) Motion plots corresponding to the two scans shown in (B) indicates that the motion performed was comparable between the two experiments.
