# Supplementary material for: Measurement and Correction of Microscopic Head Motion during Magnetic Resonance Imaging of the Brain
Source: PLoS One. 2012 Nov 7;7(11):e48088. doi: 10.1371/journal.pone.0048088 (PMC3492340; doi:10.1371/journal.pone.0048088)
Supplement: Figure S2 — Tracking data from (A) the 1.5 T and (B) the 3 T experiments. In these examples, the MPT marker was attached directly to the forehead of the subject; this differs from the results obtained at 7 T, and shown in Fig. 5, where a mouthpiece was used. With both marker attachment methods (and for all three scanners and subjects), the ballistocardiogram is visible in the z direction. In the examples shown here, respiratory motion is also clearly visible. (PDF) [file pone.0048088.s002.pdf]

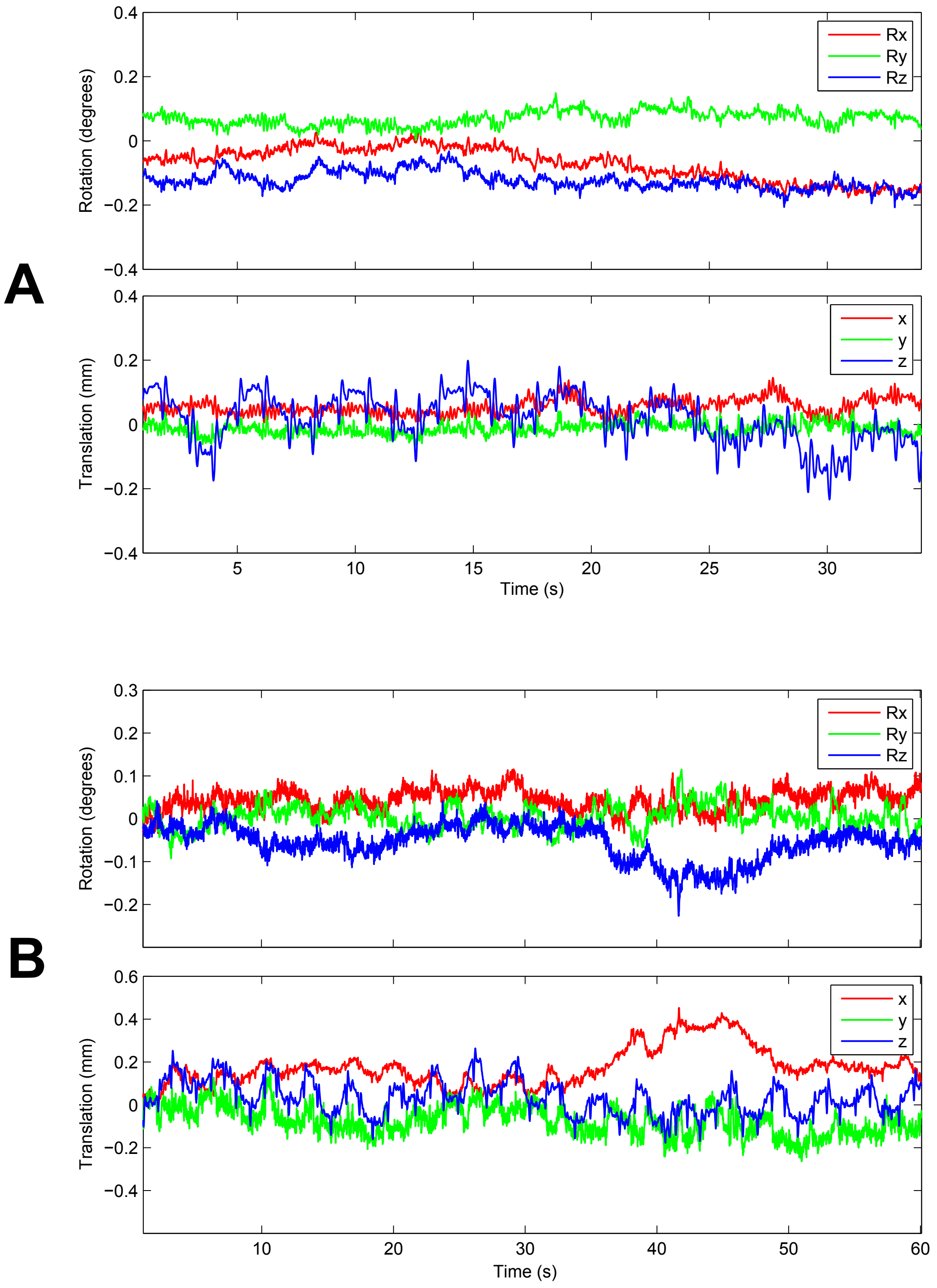

Fig. S2. Tracking data from (A) the 1.5 T and (B) the 3 T experiments. In these examples, the MPT marker was attached directly to the forehead of the subject; this differs from the results obtained at 7 T, and shown in Fig. 5, where a mouthpiece was used. With both marker attachment methods (and for all three scanners and subjects), the ballistocardiogram is visible in the z direction. In the examples shown here, respiratory motion is also clearly visible.
