# Supplementary material for: Measurement and Correction of Microscopic Head Motion during Magnetic Resonance Imaging of the Brain
Source: PLoS One. 2012 Nov 7;7(11):e48088. doi: 10.1371/journal.pone.0048088 (PMC3492340; doi:10.1371/journal.pone.0048088)
Supplement: Figure S3 — Ballistocardiograms computed using tracking data from (A) the 1.5 T and (B) the 3 T experiments (see Fig. 5 for results obtained at 7 T). In each case, the BCG appears most strongly as a shift along the head-feet direction. (PDF) [file pone.0048088.s003.pdf]

A

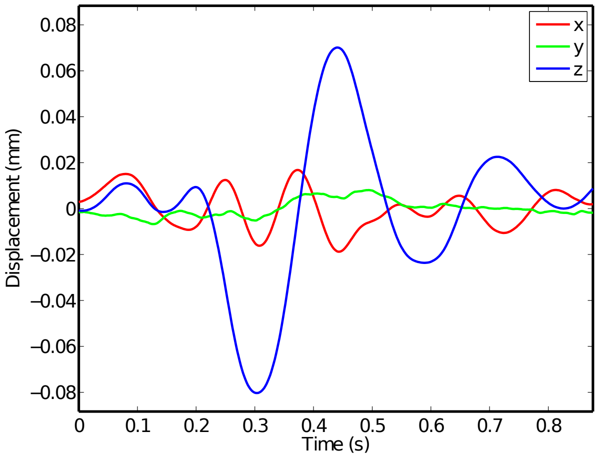

DOF	BCG mag.
x	36 µm
y	15 µm
z	150 µm
Rx	0.034 deg.
Ry	0.010 deg.
Rz	0.030 deg.

B

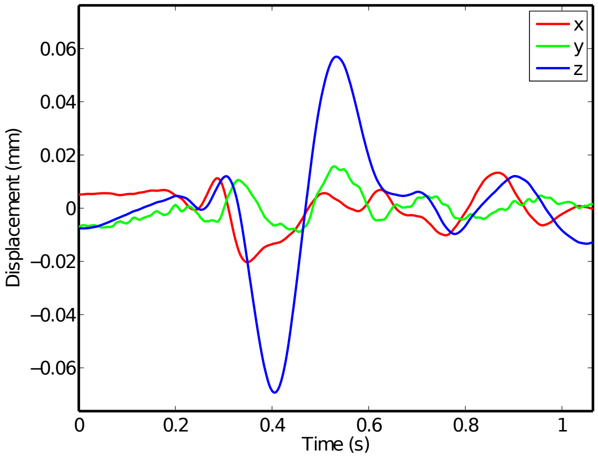

DOF	BCG mag.
x	34 µm
y	25 µm
z	126 µm
Rx	0.033 deg.
Ry	0.021 deg.
Rz	0.018 deg.

Fig. S3. Ballistocardiograms computed using tracking data from (A) the 1.5 T and (B) the 3 T experiments (see Fig. 5 for results obtained at 7 T). In each case, the effect appears most strongly as a shift along the head-feet direction.
